# Autocrine Regulation of Pulmonary Inflammation by Effector T-Cell Derived IL-10 during Infection with Respiratory Syncytial Virus

**DOI:** 10.1371/journal.ppat.1002173

**Published:** 2011-08-04

**Authors:** Jie Sun, Amber Cardani, Ashish K. Sharma, Victor E. Laubach, Robert S. Jack, Werner Müller, Thomas J. Braciale

**Affiliations:** 1 The Beirne B. Carter Center for Immunology Research, The University of Virginia, Charlottesville, Virginia, United States of America; 2 Department of Surgery, The University of Virginia, Charlottesville, Virginia, United States of America; 3 Department of Immunology, University of Greifswald, Germany; 4 Bill Ford Chair of Cellular Immunology, Faculty of Life Sciences, University of Manchester, Manchester, United Kingdom; 5 Department of Microbiology, The University of Virginia, Charlottesville, Virginia, United States of America; 6 Department of Pathology, The University of Virginia, Charlottesville, Virginia, United States of America; University of Pensylvania, United States of America

## Abstract

Respiratory syncytial virus (RSV) infection is the leading viral cause of severe lower respiratory tract illness in young infants. Clinical studies have documented that certain polymorphisms in the gene encoding the regulatory cytokine IL-10 are associated with the development of severe bronchiolitis in RSV infected infants. Here, we examined the role of IL-10 in a murine model of primary RSV infection and found that high levels of IL-10 are produced in the respiratory tract by anti-viral effector T cells at the onset of the adaptive immune response. We demonstrated that the function of the effector T cell -derived IL-10 in vivo is to limit the excess pulmonary inflammation and thereby to maintain critical lung function. We further identify a novel mechanism by which effector T cell-derived IL-10 controls excess inflammation by feedback inhibition through engagement of the IL-10 receptor on the antiviral effector T cells. Our findings suggest a potentially critical role of effector T cell-derived IL-10 in controlling disease severity in clinical RSV infection.

## Author Summary

IL-10 is a major anti-inflammatory protein that plays an essential role in regulating the balance between pathogen clearance by the immune response and immune mediated injury resulting from the immune response to pathogen infection. In this report, we demonstrate that anti-viral effector T cells, a critical cell type responsible for respiratory syncytial virus clearance, are able to produce a large quantity of IL-10. The function of IL-10 is to control the immune response in order to avoid the development of excessive pulmonary inflammation associated with the clearance of infectious virus. We further identified a likely mechanism that T cell-derived IL-10 operates to control inflammation and describe a novel potential target of IL-10 action in the RSV infected lungs. Our data thus may lay the ground for the future studies exploring the application of IL-10 in therapeutic approaches to modulate pulmonary inflammation and injury in young infants suffering severe respiratory syncytial virus induced diseases.

## Introduction

Respiratory syncytial virus (RSV) infection is the leading viral cause of upper and lower respiratory tract illness in young infants. In the USA, nearly 100% of children are infected with RSV by the age of 2–3 [1]. Approximately 1–2% of these infected children develop moderate to severe bronchiolitis [2]. The exact mechanisms underlying the development of severe pulmonary diseases in the small proportion of children remain poorly defined. Nevertheless, in both clinical studies and animal models, severe pulmonary disease induced by RSV infection is typically associated with an exaggerated inflammatory response in the lower respiratory tract, characterized by the overproduction of pro-inflammatory cytokines/chemokines and increased infiltration of inflammatory cells [3], [4], [5]. Furthermore, there is no firm correlation between disease severity and the extent of RSV replication [6], further suggesting a likely important role of the host immune response to RSV in determining disease severity. Consequently, in responding to an infectious agent like RSV with a strong potential to induce immune-mediated pathology, there is a need to finely balance the immuno-protective and immuno-pathological potential of the immune response in order to insure virus clearance without excess inflammatory injury.

IL-10 is a major regulatory cytokine with broad anti-inflammatory properties [7]. Depending on the nature of the pathogenic stimulus, many cell types including neutrophils, NK cells, macrophages, dendritic cells (DC), regulatory and effector T cells have been shown to be capable of producing IL-10 both *in vitro* and *in vivo* in response to infection [8], [9]. IL-10 is generally viewed as a negative regulator of the response of both innate and adaptive immune cells during infection particularly during persistent parasitic, bacterial, and viral infections where it can suppress pathogen clearance and/or the inflammatory response triggered by the infectious agent [8]. Recent evidence suggests that IL-10 may play an important regulatory role in acute viral infections of the respiratory tract where it inhibits the development of excess pulmonary injury in the face of normal virus clearance from the respiratory tract [10]. These findings, along with evidence of a link between a polymorphism in the IL-10 locus and the severity of bronchiolitis in infants infected with RSV [11], [12], [13], [14], prompted us to explore the role of IL-10, and in particular of IL-10 produced by virus-specific effector T-cells, in controlling pulmonary injury associated with experimental murine RSV infection.

In this study, we investigated the source and role of IL-10 during primary RSV infection. We found that high levels of this regulatory cytokine are produced simultaneously with effector cytokines in the respiratory tract at the onset of the adaptive immune response. The main cellular sources of IL-10 in the lung are RSV-specific CD4^+^ and CD8^+^ effector T-cells. We show that effector T cell-derived IL-10 during RSV infection *in vivo* acts to inhibit excess inflammation in the respiratory tract and thereby maintain critical pulmonary function in the infected host. We further provide evidence for a novel mechanism of IL-10-mediated inflammation where effector T cell-derived IL-10 acts in an autocrine manner on the effector T cells to suppress excess pulmonary inflammation induced by the anti-viral response of the effector T cells. The implications of these findings for RSV infection are discussed.

## Results

### Adaptive immune cells are the major source of IL-10 during acute RSV infection

**Figure 1:**
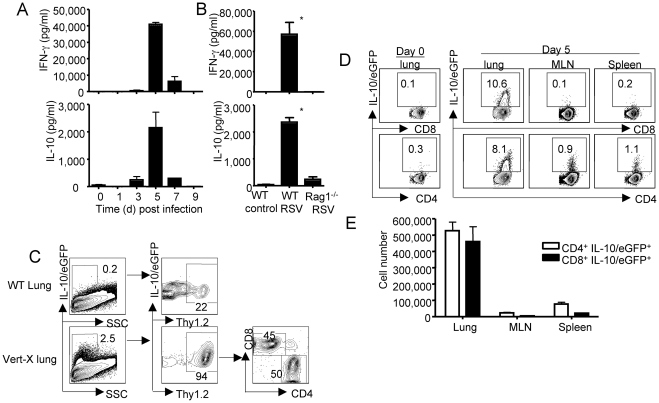
T cells are the major source of IL-10 during Respiratory Syncyial Virus infection. (A) BALB/c mice were infected with RSV. At the indicated days p.i., mice were sacrificed, and IL-10 and IFN-γ levels in the airway were measured by ELISA. (B) WT and Rag1^-/-^ mice were infected with RSV. At d5 p.i., mice were sacrificed, and IL-10 and IFN-γ levels in the airway were measured by ELISA. *P* value was determined by unpaired two-tailed Student *t* test. * indicates P< = 0.05. (C–E) Vert-X mice were infected with RSV, at d5 p.i., the expression of IL-10/eGFP by lung cells was measured by flow cytometry. (C) The phenotype of IL-10/eGFP^+^ cells in the lungs was analyzed by flow cytometry. Numbers are the percentages of cells in gated population. (D) IL-10/eGFP expression by CD8^+^ and CD4^+^ T cells from lungs, MLN or spleen of uninfected (Day 0) mice or d5 infected mice are measured by flow cytometry. Numbers are the percentages of cells in gated populations. (E) The absolute numbers of IL-10/eGFP^+^ CD8^+^ and IL-10/eGFP^+^ CD4^+^ T cells in the lungs, MLN and spleens are depicted. Data are representative of two to three independent experiments.

Because of IL-10′s documented role in controlling or inhibiting the development of excess inflammation in response to infection and injury, and due to the evidence linking expression of IL-10 to injury severity in human RSV infection, we evaluated the role of IL-10 in virus clearance and the control of pulmonary inflammation in a murine model of experimental RSV infection [8], [14]. To address this question, we first infected BALB/c mice with RSV and measured by ELISA the kinetics of IL-10 release into the bronchoalveolar lavage fluid (BALF) sampled from RSV-infected lungs. As a measure of proinflammatory cytokine release during infection we monitored in parallel the kinetics of IFN-γ production in the infected lungs. We found that minimal levels of either cytokine were detected in the BALF early during infection (day 1–3 p.i.) (Figure 1A). By day 5 p.i., a time at which RSV effector T cells have been shown to begin infiltrating into the respiratory tract [15], both IL-10 and IFN-γ levels in the BALF increased dramatically in a coordinated manner. The levels of the two cytokines then decreased progressively and both returned to background levels by day 9 p.i. (Figure 1A).

The kinetics of IL-10 and IFN-γ release into the BALF and its tight association with the influx of RSV-specific effector T cells into lungs raised the possibility that these 2 cytokines may be products of adaptive immune cells, or at least that their expression is linked to the recruitment of virus-specific adaptive immune cells into the infected lungs. To initially explore the contribution of adaptive immune (T and/or B) cells to the IL-10 response in the infected respiratory tract, we infected WT or Rag1^-/-^ mice (which lack T and B cells) with RSV and measured IL-10 and IFN-γ levels in the BALF of these mice. We found that high levels of both cytokines were released into the airways of WT but not Rag1^-/-^ mice after RSV infection (Figure 1B). Uninfected WT (Figure 1B) or Rag1^-/-^ (data not shown) mice had negligible levels of this regulatory cytokine. These data suggest that the release of both regulatory cytokine IL-10 and effector cytokine IFN-γ following infection is dependent on the adaptive immune T and/or B cells.

### T cells are the main source of IL-10 produced in the respiratory tract during RSV infection

To further define the cellular source(s) of IL-10 *in vivo* during RSV infection, we infected the IL-10/eGFP reporter mice (Vert-X) with RSV [16]. At day 5 p.i. (i.e at the peak of IL-10 detection in the BALF) we harvested lung cells and measured IL-10/eGFP expression by the liberated lung cell populations using flow cytometry. We found that, compared to the background fluorescence observed in lung cells from RSV infected WT mice, a significant percentage of lung cells from infected Vert-X mice express IL-10/eGFP (Figure 1C). More importantly we found that IL-10/eGFP expression in the Vert-X lungs was restricted almost exclusively (>90%) to Thy-1^+^ cells infiltrating the infected lungs and that the frequency of IL-10/eGFP^+^ CD4^+^ T cells among the Thy-1^+^ lymphocytes roughly equaled that of IL-10/eGFP^+^ CD8^+^ T cells (Figure 1C). These data further reinforced the view that CD4^+^ and CD8^+^ T lymphocytes are the major cellular sources of IL-10 in the lung during RSV infection. Next, we examined the IL-10/eGFP expression by CD8^+^ and CD4^+^ T cells from the uninfected (day 0) Vert-X lungs and from the lungs, the draining mediastinal lymph nodes (MLN) and the spleens of RSV infected Vert-X mice. We found that IL-10/eGFP-expressing cells are highly enriched in the RSV infected lungs, but not in the uninfected lungs nor in the MLN or the spleen of infected mice (Figure 1D, E). We also investigated the kinetics of accumulation of IL-10-expressing cells in the lungs following RSV infection and found that the IL-10-expressing cells were restricted to the acute phase of infection (Figure S1). Notably, even though the absolute number of IL-10/eGFP-expressing (IL-10 mRNA^ +^) CD8^+^ and CD4^+^ continued to increase in the lungs from d5 to d7 post infection, the in vivo release of IL-10 protein in the airway peaks at d5 post infection, which coincides with the fall in lung virus titers and so the viral antigen load in the lung. This observation is consistent with our previously reported findings [10], [17] and likely reflects dependence of IL-10 protein synthesis and release on TCR engagement and viral antigen recognition.

### IL-10 is produced in the lungs during RSV infection primarily by effector T-cells

**Figure 2:**
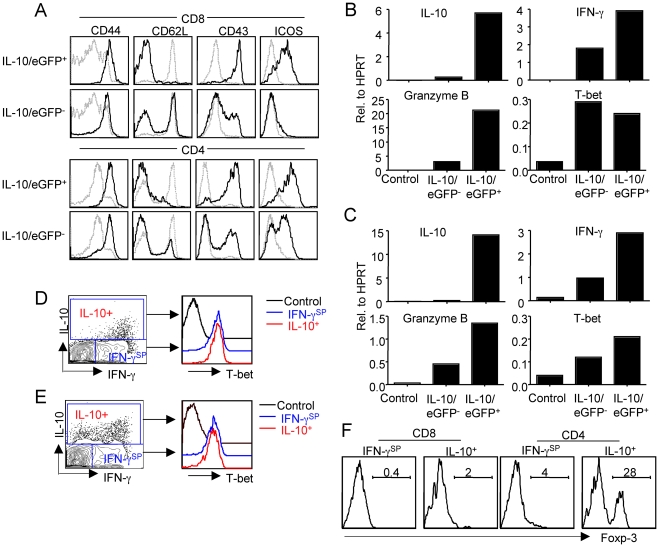
IL-10 expressing T cells are mainly type 1 effector T cells. (A), Vert-X mice were infected with RSV, At d5 p.i., the expression of CD44, CD62L, CD43 and ICOS by IL-10/eGFP^+^ or IL-10/eGFP^−^ T cells were measured by flow cytometry. Grey line, surface expression of the molecules displayed by T cells isolated from naïve lungs (Control). Black line, surface expression of the molecules displayed by IL-10/eGFP^+^ or IL-10/eGFP^−^ T cells isolated from d5 infected lungs. (B, C) Vert-X mice were infected with RSV, At d6 p.i., CD44^hi^ IL-10/eGFP^+^ cells and CD44^hi^ IL-10/eGFP^−^ T cells were sorted from infected lungs. (B) The expression of IL-10, IFN-γ, Granzyme B and T-bet genes in control (naïve) CD8^+^ T-cells, CD8^+^ CD44^hi^ IL-10/eGFP^−^ T cells and CD8^+^ CD44^hi^ IL-10/eGFP^+^ T cells was determined by quantitative RT-PCR. (C) The expression of IL-10, IFN-γ, Granzyme B and T-bet genes in control (naïve) CD4^+^ T cells, CD4^+^ CD44^hi^ IL-10/eGFP^−^ T cells and CD4^+^ CD44^hi^ IL-10/eGFP^+^ T cells was determined by quantitative RT-PCR. (D. E. F.) BALB/c mice were infected with RSV. At d5 p.i., lung cells were stimulated with PMA/Ionomycin. (D, E) The expression of IL-10, IFN-γ and T-bet by CD8^+^ T cells (D) or CD4^+^ T cells (E) was measured through ICS. (F) The expression of Foxp-3 by indicated cell population was measured by ICS. Data are representative of at least two independent experiments.

The coordinated release of IL-10 and IFN-γ in the respiratory tract suggests that the IL-10 producing T cells in the RSV infected lungs may be anti-viral effector T cells capable of producing IFN-γ as well. To address this possibility, we first examined the expression of several cell surface molecules associated with T-cell activation and/or effector differentiation by the lung T cells from uninfected Vert-X mice (control), along with lung IL-10/eGFP^+^ and IL-10/eGFP^−^ T cells from RSV infected Vert-X mice. We found that both IL-10/eGFP^+^ as well as IL-10/eGFP^−^ CD8^+^ T cells from infected Vert-X lungs express high levels of T cell activation/effector markers such as CD44, CD43, ICOS, and low levels of naïve T cell marker CD62L suggesting that, like the eGFP^−^ CD8^+^ T cells, the IL-10/eGFP^+^ CD8^+^ T cells likely represent activated effector CD8^+^ T cells (Figure 2A). Likewise, both IL-10/eGFP^+^ and IL-10/eGFP^−^ CD4^+^ T cells express higher levels of those activation/effector markers compared to control CD4^+^ T cells (Figure 2A). We also FACS-sorted IL-10/eGFP^+^ and IL-10/eGFP^−^ T cells from infected lungs and measured the gene expression of signature molecules of effector cells. We found that both CD8^+^ IL-10/eGFP^+^ cells and CD4^+^ IL-10/eGFP^+^ cells express high levels of effector molecules such as IFN-γ, Granzyme B and the type 1 effector cell lineage specific transcription factor T-bet [18], suggesting that the IL-10 expressing CD8^+^ and CD4^+^ cells are indeed type 1 effector cells (Figure 2B, C). Consistent with this idea, we observed that all IL-10-producing CD8^+^ and most IL-10-producing CD4^+^ T cells simultaneously produce IFN-γ in response to mitogenic or antigenic stimulation in vitro in the intracellular staining assay (ICS) (Figure 2D, E and data not shown). Furthermore, we analyzed by ICS assay the expression of T-bet and the regulatory T cell specific transcription factor Foxp-3 [19], in IL-10^−^ IFN-γ^+^ (IFN-γ single positive, IFN-γ^SP^) CD8^+^ T cells, IL-10^−^ IFN-γ^+^ (IFN-γ^SP^) CD4^+^ T cells , IL-10^+^ CD8^+^ T cells and IL-10^+^ CD4^+^ T cells from the infected lungs directly *ex vivo*. Consistent with the mRNA levels (Figure 2B, C), we found that IL-10 positive CD8^+^ or CD4^+^ T cells express T-bet at levels as high as the (IL-10^−^) IFN-γ single-positive CD8^+^ or CD4^+^ type 1 like effector cells (Figure 2D, E). Furthermore, most of IL-10^+^ CD8^+^ cells and the majority of the IL-10^+^ CD4^+^ T cells are Foxp-3 negative (Figure 2F). Collectively, these data suggest that the vast majority of the IL-10-expressing CD8^+^ and CD4^+^ T cells are primarily type 1 effector cells. We did however observe that a minor proportion, up to 30%, of the IL-10^+^ CD4^+^ T-cells, expressed Foxp-3. These Foxp-3^+^ CD4^+^ T cells were uniformly negative for IFN-γ production (Figure 2F and data not shown).

### IL-10 derived from effector T cells controls host morbidity without affecting viral clearance

**Figure 3:**
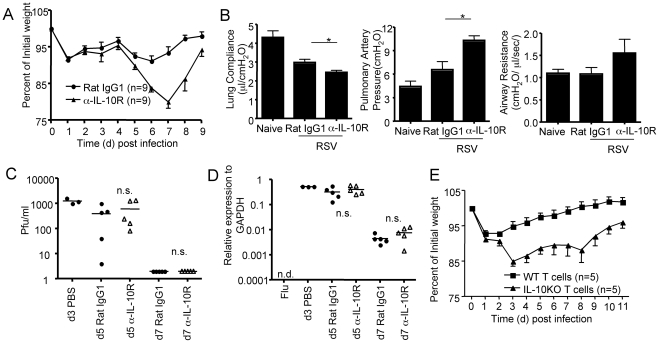
The blockade of effector T cell-derived-IL-10 enhances host morbidity without affecting viral clearance. (A-D). BALB/c mice were infected with RSV and then were either injected with Rat IgG1 control mAb or α-IL-10R blocking mAb. (A) Mouse weight loss was monitored daily following infection. (B) Pulmonary function of naïve mice, Rat IgG1 mAb treated or α-IL-10R mAb treated d7 infected mice was monitored. *P* value was determined by unpaired two-tailed Student *t* test. * indicates P< = 0.05. (C) Airway virus titers from the indicated mice were determined by plaque assay. PBS, mice were infected with RSV and received PBS. *P* value was determined by unpaired two-tailed Student *t* test. n.s.(non-significant). (D) Lung RSV gene copies from the indicated mice were determined through quantitative RT-PCR. Flu, mice were infected with influenza A/PR8 virus. *P* value was determined by unpaired two-tailed Student *t* test. n.s.(non-significant). (E) Either WT or IL-10^-/-^ naïve T cells were transferred into Rag1^-/-^ mice which were then infected with RSV. Mouse weight loss was monitored daily after infection. A, B. Pooled data from three experiments are represented. C, D, E. Pooled data from two experiments are represented.

To determine if the effector T cell-derived IL-10 had any impact on the outcome of RSV infection, we examined the effect of blockade of the IL-10 receptor (IL-10R) by *in vivo* administration of a blocking anti-IL-10Rα (α-IL-10R) mAb on virus clearance, pulmonary function and lung inflammation. We found that the administration of α-IL-10R mAb *in vivo* significantly enhanced the weight loss of RSV infected mice, particularly at day 5 and thereafter when RSV-specific adaptive immune T cells began infiltrating into the respiratory tract (Figure 3A). We also determined whether three parameters of lung function, as described in Methods, were similarly affected after blocking the action of IL-10 *in vivo*. As Figure 3B demonstrates, the blockade of IL-10 function *in vivo* leads to a significant decrease in lung compliance (LC) and increase in pulmonary artery pressure (PAP) in the treated mice at d7 post infection. In addition, although it did not reach statistical significance, airway resistance (AR) was also increased in the mice treated with α-IL-10R mAb compared to the mice administered control mAb (Figure 3B).

We next compared viral clearance in RSV infected mice which were treated with either Rat IgG1 control mAb or α-IL-10R mAb by plaque assay and by quantitative RT-PCR for RSV genome copies. As Figure 3C, and D demonstrate, mice treated with α-IL-10R mAb had cleared virus by day 7 post infection as efficiently as the mice receiving Rat IgG1 control mAb, suggesting that the blockade of IL-10R signaling *in vivo* does not alter the kinetics of viral clearance in the lung during RSV infection. These results collectively demonstrated that the blockade of IL-10 function *in vivo* leads to enhanced host diseases without affecting viral clearance. Although we cannot exclude formally the contribution of IL-10 derived from the small fraction of Thy-1^−^/CD3^−^ IL-10^+^ cells in the infected lungs (Figure 1C) to the control of inflammation, our results strongly suggest that effector T-cells are the major source and the most important source of this regulatory cytokine.

To determine the role of T cell-derived IL-10 in restraining host morbidity, we transferred naive WT or IL-10^-/-^ Thy1^+^ cells (including both CD4^+^ and CD8^+^ T cells) into Rag1^-/-^ mice and infected the recipient animals with RSV. We found that Rag1^-/-^ mice reconstituted with IL-10^-/-^ T cells had increased weight loss compared to Rag1^-/-^ mice reconstituted with WT T cells following RSV infection (Figure 3E). These data suggested that IL-10 derived from T cells themselves is able to control host morbidity in response to RSV infection.

### Blocking the action of IL-10 in vivo results in increased pulmonary inflammation

**Figure 4:**
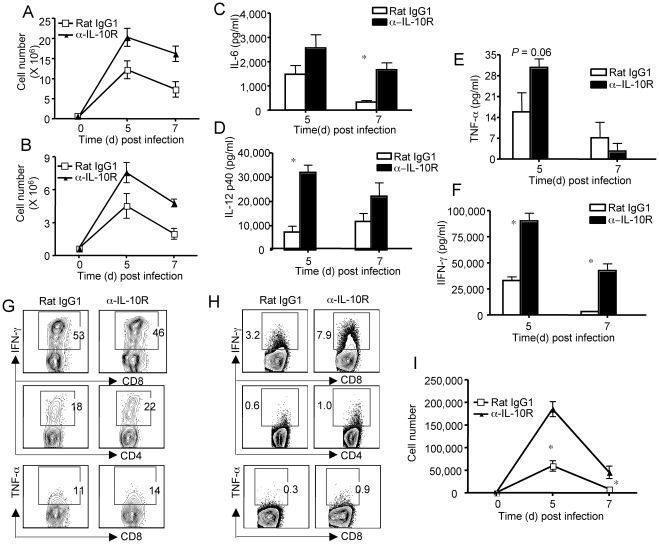
The blockade of effector T cell-derived-IL-10 leads to enhanced pulmonary innate and adaptive inflammation. (A–I) BALB/c mice were infected with RSV and treated with either Rat IgG1 control mAb or α-IL-10R blocking mAb. *P* value was determined by unpaired two-tailed Student *t* test. * indicates P< = 0.05. (A, B) At the indicated days p.i., lung inflammatory monocytes (A) and neutrophils (B) were determined by flow cytometry. (C–F) At indicated days p.i., airway IL-6 (C), IL-12 p40 (D), TNF-α (E), IFN-γ (F) were determined by ELISA. (G) At d5 p.i., lung cells were collected and restimulated with PMA/Ionomycin. The production of IFN-γ by CD4^+^ and CD8^+^ T cells and TNF-α by CD8^+^ T cells was measured by ICS. Numbers are the percentages of cells in gated populations. (H. I) Mice were injected with monensin to block the *in vivo* release of cytokine. Lung cells were then collected and the in vivo production of IFN-γ, and TNF-α determined by ICS. (H) The percentages of IFN-γ^+^ cells in gated lung CD8^+^ or CD4^+^ T cells and TNF-α^+^ cells in lung CD8^+^ T cells at d5 p.i. are shown. Numbers are the percentages of cells in gated populations. (I) The numbers of IFN-γ^+^ lung CD8^+^ T cells at indicated days p.i. are shown. Data are representative of at least three independent experiments.

We next examined whether the blockade of IL-10 action *in vivo* leads to enhanced pulmonary inflammation following RSV infection. We first determined the impact of IL-10R blockade on the recruitment of innate inflammatory cells to the lung. We found that IL-10R blockade resulted in a substantial increase in the number of proinflammatory monocytic cells and notably neutrophils infiltrating the infected lungs (Figure 4A, B). This increase in inflammatory monocyte and neutrophil infiltration is accompanied by enhanced release of pro-inflammatory cytokines including IL-12/23 p40, IL-6, TNF-α and IFN-γ into the airways of α-IL-10R mAb-treated mice compared to control mAb-treated mice (Figure 4C–F). Taken together, these data suggest that a critical function of T-cell-derived IL-10 *in vivo* during RSV infection is to prevent the development of excessive pulmonary inflammation in the infected respiratory tract associated with virus infection and the host innate/adaptive immune response and to retain essential lung function in the infected host without inhibiting virus clearance.

### IL-10R blockade in vivo results in enhanced proinflammatory activity of effector T cells

Along with their role in virus clearance [20], effector T cells have been shown to significantly contribute to lung inflammation and host morbidity in the murine model of primary RSV infection [3], [20], [21]. The enhanced morbidity, excess pulmonary inflammation/injury and altered pulmonary function observed with IL-10R blockade during RSV infection may reflect a normal function of IL-10 in regulating the induction, expansion and/or effector activity of effector T cells responding in the lungs to infection. We first investigated the impact of IL-10R blockade *in vivo* on the induction (in the draining MLN) and migration of effector T cells to the lung. Somewhat unexpectedly, the numbers of activated/effector CD8^+^ or CD4^+^ T cells in the infected lungs were comparable in mice treated either with control Rat IgG1 mAb or α-IL-10R mAb (Figure S2). Furthermore, effector T cells from either control or α-IL-10R-treated mice had comparable capability to produce IFN-γ and TNF-α in response to mitogenic or antigenic stimulation (Figure 4G and data not shown), suggesting that IL-10 does not affect the differentiation of T cells into RSV specific effector T-cells of the Th1 or Tc1 lineage.

In view of the elevated levels of lung proinflammatory cytokines, in particular IFN-γ, we next explored the impact of IL-10R blockade on the in vivo frequency and cytokine profile of virus-specific T cells responding in the infected respiratory tract. For this purpose we chose IFN-γ and TNF-α as representative effector cytokines and used the the *in vivo* ICS assay [22] to measure their release by CD8^+^ and CD4^+^ T cells in the respiratory tract. We found that blockade of IL-10 function *in vivo* triggered an increase in the percentage/number of CD4^+^ and particularly CD8^+^ T cells producing IFN-γ *in vivo* (Figure 4H, I). In addition, the *in vivo* production of TNF-α by CD8^+^ T cells was also modestly increased following IL-10R blockade during RSV infection (Figure 4H). The blockade of IL-10 function *in vivo* also leads to increased production of IFN-γ on a per cell basis (Figure S3). These data demonstrate that this effector T cell-derived IL-10 can act *in vivo* to suppress the production of proinflammatory mediators by effector T cells responding in the respiratory tract of virus infection.

### Autocrine regulation of IL-10 to restrain host inflammation and morbidity

**Figure 5:**
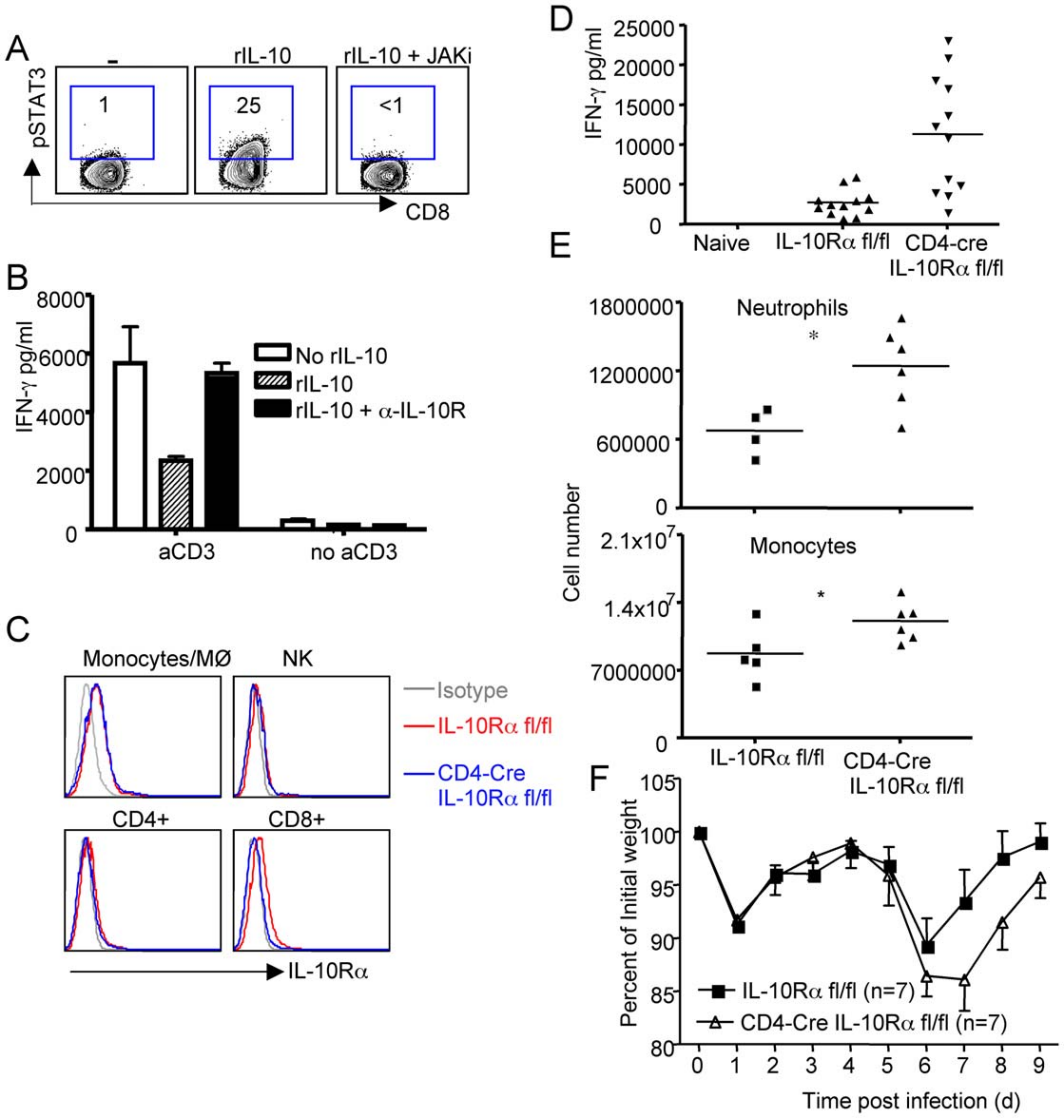
Autocrine regulation of pulmonary inflammation by effector T cell-derived IL-10 during RSV infection. (A, B). BALB/c mice were infected with RSV. At d7 p.i., Lung CD8^+^ cells were purified and stimulated as indicated *in vitro*. (A) CD8^+^ T cells were stimulated with rIL-10 in the absence or presence of JAK inhibitor. STAT3 phophorylation was determined by ICS. (B) CD8^+^ T cells were left un-stimulated or stimulated with plate-bound a-CD3 overnight in the absence or presence of rIL-10 or rIL-10 plus α-IL-10R. IFN-γ release to the medium was determined by ELISA. (C – F) IL-10Rα fl/fl mice or CD4-Cre^+^ IL-10Rα fl/fl mice were infected with RSV. (C) The expression of IL-10Rα in lung monocytes, NK cells, CD4^+^ and CD8^+^ T cells at d6 post infection was determined by flow cytometry. (D) IFN-γ levels in the BALF were determined by ELISA. *P* value was determined by unpaired two-tailed Student *t* test. * indicates P< = 0.05. (E) Lung infiltrated neutrophils and inflammatory monocytes were determined by flow cytometry. *P* value was determined by unpaired two-tailed Student *t* test. * indicates P< = 0.05. (F) Mouse weight loss was monitored daily following infection. A, B, C. Data are representative of at least two independent experiments. D, E .Pooled data from four experiments are represented. F. Pooled data from three experiments are represented.

Cells of the myeloid, monocyte/macrophage/dendritic cell lineage are believed to be the major targets of IL-10 [7]. Therefore IL-10 would most likely be expected to diminish the effector activity of effector T cells *in vivo* by inhibiting the APC function of these inflammatory mononuclear cells infiltrating the infected lungs. Alternatively, the T-cell-derived IL-10 could also act in an autocrine fashion to suppress the activation/stimulation of CD8^+^ and CD4^+^ lung effector T cells through the engagement of the IL-10R on these T-cells. To explore this latter possibility, we first isolated CD8^+^ effector T cells from RSV infected lungs and examined whether they can respond to IL-10. As Figure 5A demonstrates, we found that CD8^+^ effector T cells isolated from infected lungs are able to respond to IL-10 treatment by phosphorylating STAT-3. Furthermore, IL-10 is able to inhibit the release of IFN-γ by CD8^+^ effector T cells in response to CD3 stimulation in the absence of antigen presenting cells (Figure 5B). Collectively, these data demonstrated that IL-10 is able to signal to effector T cells and suppress their proinflammatory activity. To directly explore *in vivo* the possible contribution of an autocrine mechanism of IL-10 action during RSV infection, we examined the response to primary RSV infection of mice with a conditional deletion of the IL-10Rα gene selectively in CD4^+^ and CD8^+^ T cells [23]. We confirmed that IL-10Rα was selectively deleted in T cells, particularly CD8^+^ T cells in the lung but not in lung monocytes/macrophages or NK cells etc (Figure 5C and data not shown). Notably, compared to CD8^+^ effector T cells, we failed to detect significant IL-10Rα expression in CD4^+^ effector T cells (Figure 5C). Importantly, following RSV infection, the deletion of IL-10Rα in T cells resulted in dramatically increased release of effector T cells derived cytokine IFN-γ into the airway (Figure 5D). Interestingly, the deletion of IL-10Rα in T cells also resulted in significantly increased infiltration of neutrophils and monocytes (but not T cells) into the infected lungs (Figure 5D, and Figure S4), suggesting a role for enhanced adaptive immune-mediated inflammation in promoting neutrophil and monocyte infiltration to the lung. Consistent with the finding of increased inflammation in the lung, we observed that conditional deletion of the IL-10Rα in T cells resulted in increased weight loss and delayed recovery following RSV infection of the knockout mice (Figure 5E).

## Discussion

In this report, we examined the *in vivo* production, cellular sources and function of the regulatory cytokine IL-10 during acute pulmonary RSV infection. Importantly, the primary source of IL-10 is the anti-viral CD4^+^ and CD8^+^ effector T cells recruited to the RSV infected lungs. We demonstrate that this effector T cell-derived IL-10 plays a critical role in preventing excess inflammation during the innate and particularly the adaptive immune response to RSV. In the absence of IL-10 signaling, an enhanced inflammatory response and a concomitant alteration in pulmonary function ensues. Thus, IL-10 may play a critical role in maintaining lung function during infection. Of note, our findings suggest that effector T cells are not only the major source of IL-10 in the infected lungs but may also serve as important cellular targets for the action of this regulatory cytokine, reflecting a novel autocrine pathway for the action of IL-10 during infection.

Several lines of evidence in the current study implicate effector T cells as the primary source of IL-10 observed in the RSV infected respiratory tract in this model. Similar to our recently reported findings in the murine influenza infection model [10], we noted minimal IL-10 secretion in RSV infected Rag1^-/-^ mice and localized IL-10 production by effector T cells in the infected lungs using the IL-10 reporter Vert-X mice for infection. However, we cannot formally exclude a major contribution of the small percentage (2%–5%) of Thy-1^– (negative)^ cells detected to the amelioration of pathology. In this case, however, we demonstrated that IL-10 derived solely from T-cells are sufficient to control disease development during RSV infection (Figure 3E) which also implicated T-cell-derived IL-10 in the control of excess pulmonary inflammation. The IL-10-producing effector T cells are, as we observed in this report, IL-4 and GATA-3 negative (data not shown) and thus show few features characteristic of type 2 lineage of T cells; but rather are T-bet positive type 1 effectors. Interestingly, we observed a minor fraction of T-bet^+^ IL-10 producers which co-express Foxp-3, a cell population that has been recently described in other systems [24]. Of note, CD25^+^ T regulatory cells have been recently demonstrated to modulate RSV specific CD8^+^ T cell responses and pulmonary inflammation during experimental RSV infection [25], [26], [27]. How these Tregs interact with the IL-10 secreting effector T cells responding in the respiratory tract to RSV infection to control excess inflammation will require further investigation.

It is of interest that RSV infection induced a lower level of IL-10 release into the infected airways relative to the release of effector T cells derived pro-inflammatory effector cytokine IFN-γ than detected in influenza infected lungs. The explanation for this discrepancy is unclear, but this could reflect an as yet unappreciated mechanism by which RSV infection results in an imbalance in the expression of pro-inflammatory (e.g. IFN-γ and regulatory (e.g. IL-10) cytokines by effector T-cells resulting in exaggerated inflammation/injury profile in the infected lungs out of proportion to the degree of RSV replication in the infected lungs.

Multiple lines of evidence both from human studies and murine models implicate myeloid cells of the monocyte/macrophage/dendritic cell lineage as the major targets of IL-10 action *in vitro* and (in model systems) *in vivo* [7], [9], [23], [28], [29]. Thus results from the current study suggest that effector T cell-derived IL-10 can act directly on these inflammatory mononuclear cells infiltrating the RSV infected lungs to decrease production of pro-inflammatory cytokines/chemokines by these inflammatory mononuclear cells as well as to modulate the expression of costimulatory ligands (e.g. CD40, 80, 86 etc.) and T-cell stimulatory cytokines (e.g. IL-12 etc.) [28]. The latter effects of IL-10 would be expected to diminish the efficiency of effector T cell triggering in response to contact with these APC populations and thus down regulating the effector activity of T cells. While our results are consistent with such a mechanism, our findings on the impact of conditional deletion of the IL-10Rα selectively in T-cells suggests the novel possibility that the effector T cell-derived IL-10 may also act in an autocrine fashion to regulate effector T cell activity directly. Of note, using animals deficient in IL-10Rβ chain, several recent reports have provided evidence that IL-10 may act directly on and suppress the function of T cells including Treg cells [30] and CD4^+^ T cells directly *in vivo* during acute LCMV infection [31] as well as inhibit robust memory CD8^+^ T cell development [32]. It should be noted, however, that IL-10Rβ is the common subunit for receptors recognizing several other cytokines including IL-22, IL-26 and IL-28 etc [33]. Our results using the conditional deletion of the IL-10 receptor a chain, which is unique to IL-10, firmly establish that effector T cells are able to respond to IL-10 directly *in vivo* during infection. Furthermore, we described a novel autocirne function of IL-10; that effector T cell-derived IL-10 is able to signal back to effector T cells, especially CD8^+^ effector T cells, to restrict the proinflammatory cytokine production by these effector T-cells. Importantly, this autocrine regulatory function of IL-10 acting on effector T cells modulates pulmonary inflammation and thereby results in diminished host morbidity. A similar autocrine mechanism has been reported recently for regulation of IL-10 producing macrophages during endotoxin challenge [23], [29]. The mechanisms through which IL-10R engagement on activated effector T cells acts to suppress effector T cell functions at sites of infection are currently under investigation.

IL-10 has been detected both in respiratory tract secretions and in the serum of infants and young children during the acute phase of RSV infection [34]. Although controversial [14], [35], several lines of evidence suggest that the level of IL-10 secretion may correlate inversely with disease severity [12], [13]. This may be particularly evident among children hospitalized for RSV infection where those children with symptoms of severe RSV bronchiolitis requiring mechanical ventilation express lower levels of IL-10 than hospitalized children with less severe disease [13]. Similarly, a recent study reported lower IL-10 levels in stimulated cord blood of children who were hospitalized for RSV infection before 6 months of age than in cord blood of infected infants who were treated as outpatients [36]. Furthermore, homozygosity for certain IL-10 alleles correlates with a higher risk of severe RSV bronchiolitis [12]. Such studies point to the importance of IL-10 in controlling the severity of acute infection with RSV.

The sources of IL-10 during acute primary RSV infection have not been clearly defined. While various cell types have been implicated as the source of this IL-10 [34], [37], [38], [39], [40], our results point to effector T cells as a potential major source of IL-10 produced during the acute phase of RSV infection. In this connection, we did observe a small increase of IL-10 released into the airways (BAL fluid) prior to the dramatic increase in IL-10 production observed in the respiratory tract at the time of effector T cell infiltration at day 5 post RSV infection. Thus, while one or more additional cell types may contribute to the pool of IL-10 observed during acute RSV infection, our results suggest that effector T-cells (both CD4^+^ and CD8^+^) may be a major source of IL-10 during human infection. Therefore the contribution of IL-10 from effector T cells should be rigorously evaluated in future studies of human RSV infection. Our analysis also suggests that the RSV specific effector T cells in the infected lungs serve as an important target of IL-10 action. Thus, the development and extent of immune mediated pathology in RSV infection may not only be linked to the level of IL-10 production by effector T cells (and potentially other cell types in the infected lungs) but also dependent upon the effectiveness of IL-10 signaling through the IL-10R on effector T cells leading to modulation of effector T cell function.

In summary, we have discovered a critical role of effector T cell-derived IL-10 in controlling the pulmonary inflammation and function during RSV infection. Furthermore, we established a previously unrecognized autocrine function of IL-10 in controlling proinflammatory activity of anti-viral effector cells. Our findings thus provide a cellular and mechanistic link to earlier clinical studies which implicate IL-10 in the pathogenesis of RSV disease and may provide the groundwork for future studies examining IL-10 as a therapeutic option in the treatment of RSV induced bronchiolitis in young infants.

## Materials and Methods

### Mice and infection

BALB/c mice were purchased from Taconic Farms and Rag1-/- mice were purchased from The Jackson Laboratories. IL-10-eGFP reporter mice (Vert-X) were obtained from C.L. Karp from Children Hospital of Cincinnati. IL-10^-/-^ mice were obtained from T. A. Wynn from NIAID, NIH. The T cell conditional IL-10R**α** knock-out mice were generated through crossing IL-10R**α** fl/fl mice to CD4-cre transgenic mice [23]. All mice were housed in a specific pathogen-free environment and all animal experiments were performed in accordance with protocols approved by the University of Virginia Animal Care and Use Committee. The A2 strain of RSV (obtained from P. L. Collins from NIAID, NIH) was grown in HEp-2 cells (ATCC) and titered for infectivity. We infected 10–12 week old BALB/c mice with a dose of ∼1–1.2×10^7^ pfu RSV in serum-free Iscove's medium (Invitrogen) intranasally after anesthesia with methyl ether (Matix Scientific). For the conditional IL-10R**α** mice infection (B6 background), we infected 9–12 week transgenic mice with a dose of ∼2×10^7^ pfu RSV in serum-free Iscove's medium after anesthesia with ketamine and xylazine.

### Ethics statement

This study was carried out in strict accordance with the Animal Welfare Act (Public Law 91-579) and the recommendations in the Guide for the Care and Use of Laboratory Animals of the National Institutes of Health (OLAW/NIH, 2002). The protocol was approved by the University of Virginia Animal Care and Use Committee (ACUC, Protocol Number: 2230).

### T cell transfer and infection

We purified WT or IL-10-/- Thy1 positive T cells (including both CD4^+^ and CD8^+^ cells) from spleen and lymph nodes by MACS separation. 15 million T cells were then transferred into Rag1-/- mice i.v. 1 week after transfer, the recipient mice were infected with RSV.

### Bronchoalveolar lavage fluid (BALF) for virus and cytokine determination

We obtained BALF by flushing the airway multiple times with a single use of 500 µl sterile PBS through a cannula attached to a syringe. Cells were recovered from the suspension by centrifugation at 4°C. Supernatants were collected and stored at –80°C until use for the cytokine and virus determinations. Cytokines were measured by ELISA (BD Biosciences) according to the manufacturer manuals. The viral titer in the BALF was determined through the plaque assay in serially diluted Hep-2 cell cultures.

### Quantitative reverse-transcription PCR

To measure RSV-L gene expression, we isolated RNA from the infected lungs via Trizol (Invitrogen) and treated it with DNase I (Invitrogen). We used random primers (Invitrogen) and Superscript II (Invitrogen) to synthesize first-strand complementary DNAs from equivalent amounts of RNA from each sample. We performed real-time RT-PCR in a 7000 Real-Time PCR System (Applied Biosystems) with SYBR Green PCR Master Mix (Applied Biosystems). The sequence of RSV-L gene primers was previously reported [41]. Data were generated by the comparative threshold cycle (*ΔC_T_*) method by normalizing to GAPDH. For experiments to measure host genes in effector T cells from infected Vert-X mice. We isolated CD44^hi^ IL-10/eGFP^+^ or CD44^hi^ IL-10/eGFP^-^ CD8^+^ T cells and CD44^hi^ IL-10/eGFP^+^ or CD44^hi^ IL-10/eGFP^-^ CD4^+^ T cells by FACS-sorting. We then isolated RNA, synthesized cDNA and performed real-time RT-PCR as described above. The sequences of the primers are available upon request.

### Intracellular cytokine staining (ICS)

Lung single cell suspensions were generated as described [42]. Lung cells were subsequently re-stimulated with either PMA (100 ng/ml) and ionomycin (1 µg/ml) in the presence of Golgi-Stop (1 µl/ml) for 5–6 h. Then cells were fixed and permeablized using the Cyto-Fix and Perm-Wash system (BD Biosciences). Cell surface CD4 and CD8 and intracellular IL-10 and IFN-γ were stained accordingly. Measurement of IL-10 and IFN-γ producing cells *in vivo* was based on a previously described protocol with modifications [22]. Briefly, at indicated days post RSV infection mice were injected i.v. with 500 µl of a PBS solution containing 500 µg Monensin (Sigma-Aldrich) 6 h before harvesting. Lung single cell suspensions were prepared in the presence of monensin. Cells were then fixed and permeablized and intracellular IL-10 and IFN-γ staining was as described [43].

### Interleukin-10 receptor–specific monoclonal antibody administration in vivo

α-IL-10R blocking mAb (clone 1B1.3A) and isotype control Rat IgG1 mAb were obtained from Schering-plough Biopharma and Bio-express. We achieved IL-10 signaling blockade *in vivo* by injecting α-IL-10R blocking mAb on day 1 (0.75 mg intraperitoneally in 500 µl), day 3 (0.15 mg intranasally in 40 µl) and day 4 (0.75 mg intraperitoneally in 500 µl).

### Measurement of pulmonary function

The functional properties of the lung i.e. the airway and vascular mechanics are characterized by different lung function parameters, including the pulmonary compliance, the airway resistance and pulmonary artery pressure. The airway resistance is an index for the resistive forces against the airflow in the airways and depends on the diameter and length of the airways. The airway resistance can be calculated from the relation between transpulmonary pressure and airflow velocity. The airway resistance increases as a consequence of narrowing of the airways due to bronchoconstriction or obstructive processes, e.g. bronchial edema or enhanced mucus deposition. The pulmonary compliance is a marker for the functional stiffness of the lung and can be calculated from the relation between tidal volume and transpulmonary pressure. The pulmonary compliance decreases during restrictive pathological processes e.g. atelectasis, fibrosis, pulmonary edema or disturbed surfactant secretion. The increase in pulmonary artery pressure is an index of vasoconstriction of lung and represents an underlying pathophysiology resulting in pulmonary edema or changes in pulmonary vascular resistance. We measured the lung function using a buffer-perfused mouse lung system (Hugo Sachs Elektronik) as previously described [44]. Briefly, at day 7 post RSV infection, mice were anesthetized with ketamine and xylazine and ventilated with room air at 100 strokes/min with a tidal volume of 7 ml/g body weight with a positive end expiratory pressure of 2 cm H_2_O using the MINIVENT mouse ventilator (Hugo Sachs Elektronik). The pulmonary artery was cannulated via the right ventricle, and the left ventricle was immediately tube-vented through a small incision at the apex of the heart. The lungs were then perfused at a constant flow of 60 µl·g body wt–1·min–1 with Krebs-Henseleit buffer containing 2% albumin, 0.1% glucose, and 0.3% HEPES. The perfusate buffer and isolated lungs were maintained at 37°C throughout the experiment. Isolated lungs were allowed to equilibrate on the apparatus during a 5-min stabilization period. After equilibration, data were recorded for an additional 10 minutes. Hemodynamic and pulmonary parameters were continuously recorded during this period by the PULMODYN data acquisition system (Hugo Sachs Elektronik).

### STAT3 staining and inhibition of cytokine release of effector T cells

Effector T cells were purified from RSV infected lung at d7 p.i. and resuspended in complete media. Then T cells were stimulated with 20 ng/ml rIL-10 (eBioscience) in the presence or absence of JAK inhibitor (JAK inhibitor I, EMD Biosciences) and 15 min later the phosphorylation of STAT3 was determined through ICS following previousely described protocols [10]. To measure the inhibition of IFN-γ release by IL-10, we stimulated purified T cells (5×10^5^ cells/ml) with plate-bound α-CD3 (100 µl of 0.1 µg/ml α-CD3 for 4 h at 37°C) overnight. Then the supernatant of the culture were collected and IFN-γ concentration were determined through ELISA.

### FACS analysis

All FACS antibodies are purchased from BD Biosciences or eBioscience. The dilution of surface staining antibodies was 1 in 200 and the dilution of intracellular staining antibodies was 1 in 100. After antibody staining, we examined cells using a six-color FACS-Canto system (BD Biosciences) and the data were analyzed by FlowJo software (Treestar). We characterized the various cell types according to their phenotypes as follows: neutrophils (Ly6G^+^CD11b^+^Ly6c^–^), monocytic cell lineage (Ly6G^–^CD11b^+^Ly6c^+^), natural killer cells (NK1.1^+^CD3^−^), CD8^+^ T lymphocytes (Thy1^+^CD8^+^) and CD4^+^ T lymphocytes (Thy1^+^CD4^+^).

### Statistical analyses

Data are means ± SEM. We used two-tailed Student's *t* test for statistical analyses. We considered all *P* values >0.05 not to be significant.

## Supporting Information

Figure S1 **Kinetics of IL-10 expressing T cells in the lung.** Vert-X mice were infected with RSV. At indicated days p.i., the percentage (A) or total numbers (B, C) of IL-10/eGFP^+^ CD8^+^ (B) or CD4^+^ (C) cells were determined by flow cytometry. Numbers are the percentages of cells in gated populations. Data are representative of two to three independent experiments.
(TIF)

Figure S2 **IL-10R blockade** ***in vivo*** **does not alter the magnitude of effector T cell infiltration to the lung.** (A, B) BALB/c mice were infected with RSV and treated either with Rat IgG1 control mAb or α-IL-10R blocking mAb. A, B, At the indicated days p.i., lung CD8^+^ M2_82–90_ tetramer^+^ cells (A) or CD4^+^ cells were determined by flow cytometry. Data are representative of three independent experiments.
(TIF)

Figure S3 **IL-10R blockade in vivo results in increased production of IFN-γ in per cell base.** BALB/c mice were infected with RSV and treated either with Rat IgG1 control mAb or α-IL-10R blocking mAb. Infected mice were injected with monensin to block the *in vivo* release of cytokine. Then lung cells were collected and the in vivo production of IFN-γ was determined through ICS. The mean fluorescence intensity of IFN-γ is depicted. Data are representative of three independent experiments.
(TIF)

Figure S4 **IL-10Rα deletion in T cells minimally affects effector T cell infiltration to the lung.** (A, B) IL-10Rα fl/fl mice or CD4-Cre^+^ IL-10R fl/fl mice were infected with RSV. The numbers of total CD4^+^ T cells (A) or CD8^+^ (B) were determined by flow cytometry. *P* value was determined by unpaired two-tailed Student *t* test. n.s. non-significant. Data are representative of two independent experiments.
(TIF)
