# Supplementary figures and images for: Autocrine Regulation of Pulmonary Inflammation by Effector T-Cell Derived IL-10 during Infection with Respiratory Syncytial Virus
Source: PLoS Pathog. 2011 Aug 4;7(8):e1002173. doi: 10.1371/journal.ppat.1002173 (PMC3150291; doi:10.1371/journal.ppat.1002173)

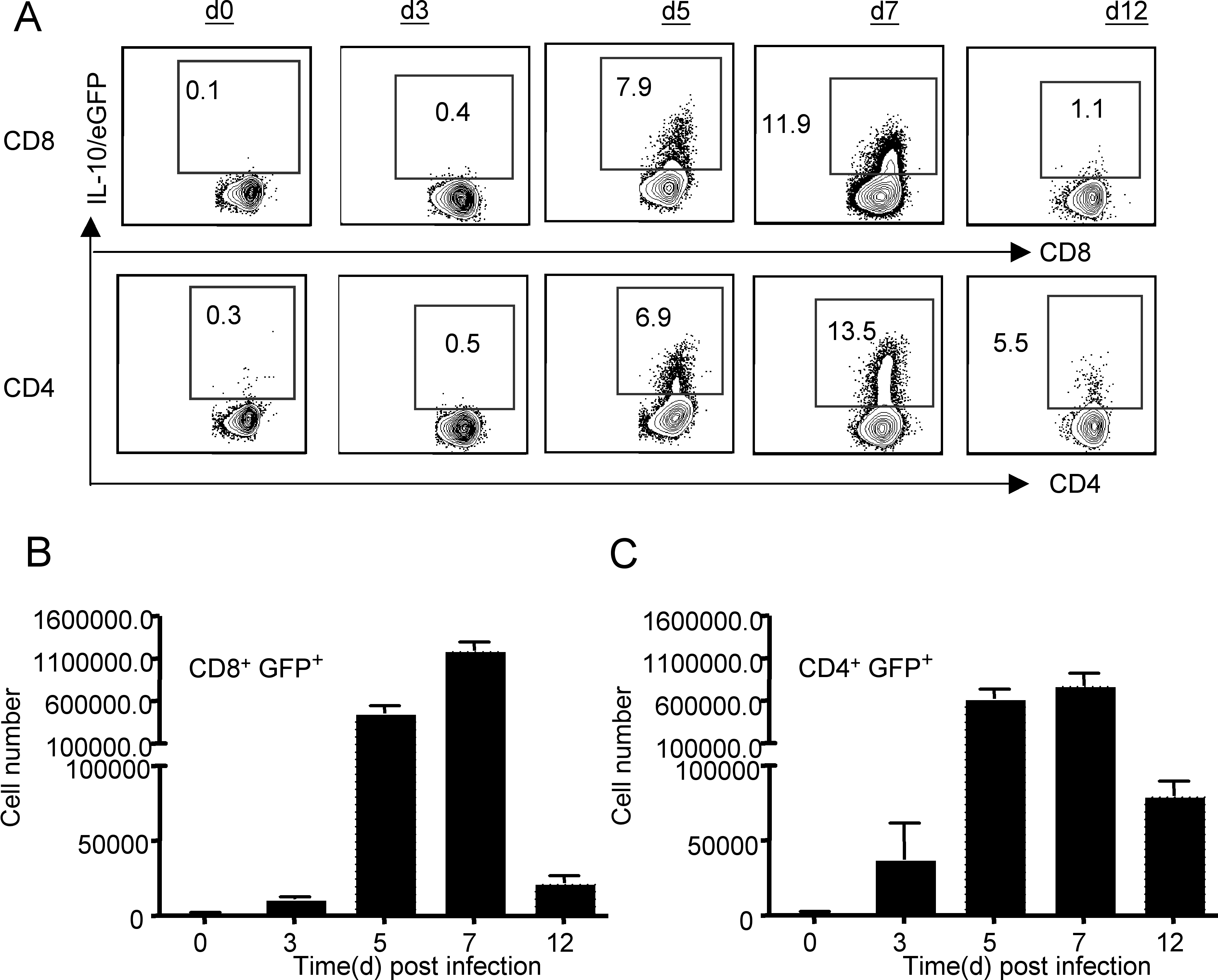

Supplement: Figure S1 — Kinetics of IL-10 expressing T cells in the lung. Vert-X mice were infected with RSV. At indicated days p.i., the percentage (A) or total numbers (B, C) of IL-10/eGFP+ CD8+ (B) or CD4+ (C) cells were determined by flow cytometry. Numbers are the percentages of cells in gated populations. Data are representative of two to three independent experiments. (TIF) [file ppat.1002173.s001.tif]

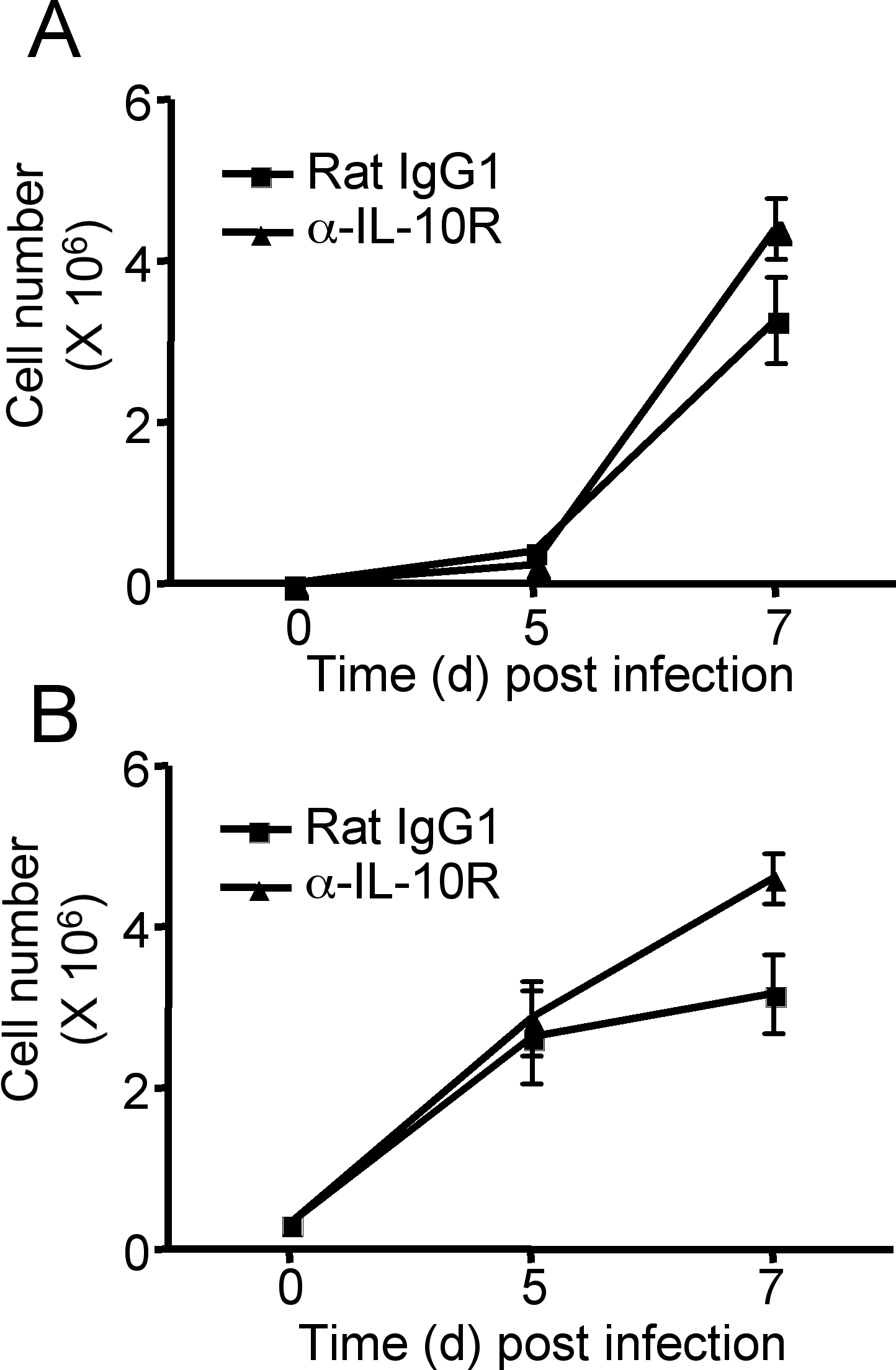

Supplement: Figure S2 — IL-10R blockade in vivo does not alter the magnitude of effector T cell infiltration to the lung. (A, B) BALB/c mice were infected with RSV and treated either with Rat IgG1 control mAb or α-IL-10R blocking mAb. A, B, At the indicated days p.i., lung CD8+ M282–90 tetramer+ cells (A) or CD4+ cells were determined by flow cytometry. Data are representative of three independent experiments. (TIF) [file ppat.1002173.s002.tif]

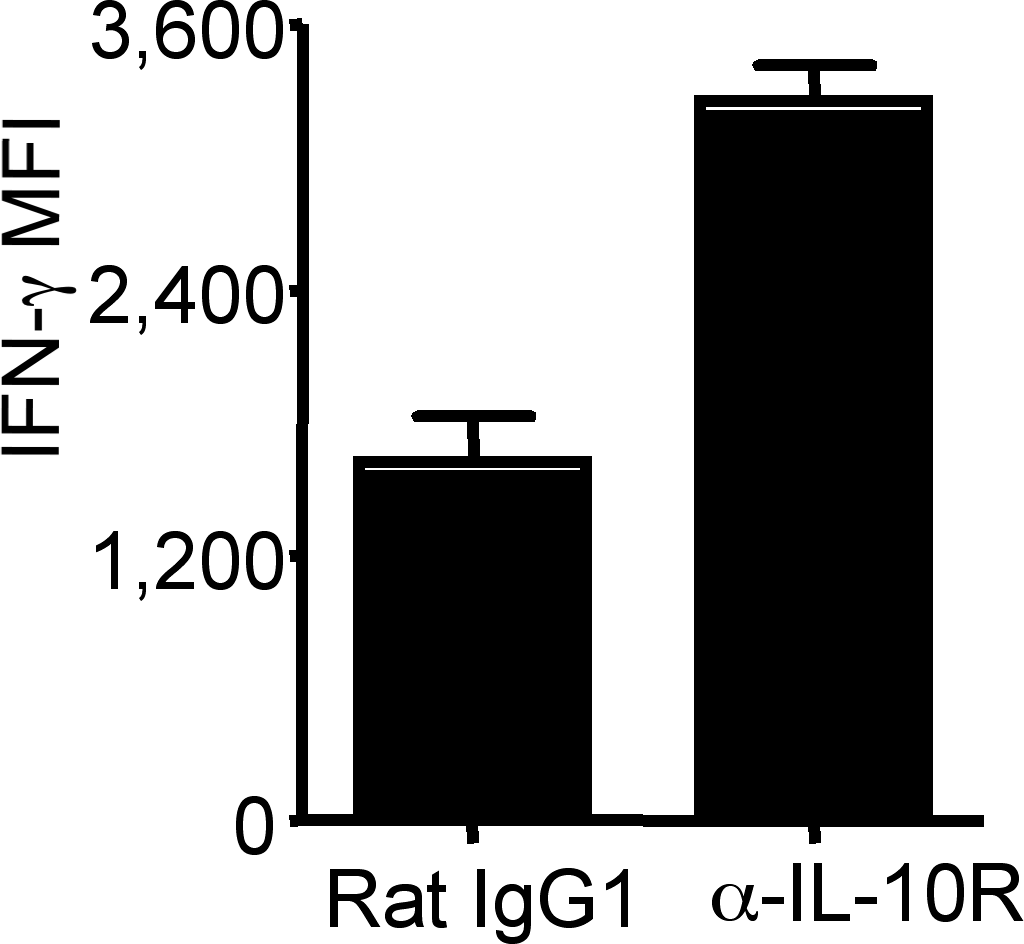

Supplement: Figure S3 — IL-10R blockade in vivo results in increased production of IFN-γ in per cell base. BALB/c mice were infected with RSV and treated either with Rat IgG1 control mAb or α-IL-10R blocking mAb. Infected mice were injected with monensin to block the in vivo release of cytokine. Then lung cells were collected and the in vivo production of IFN-γ was determined through ICS. The mean fluorescence intensity of IFN-γ is depicted. Data are representative of three independent experiments. (TIF) [file ppat.1002173.s003.tif]

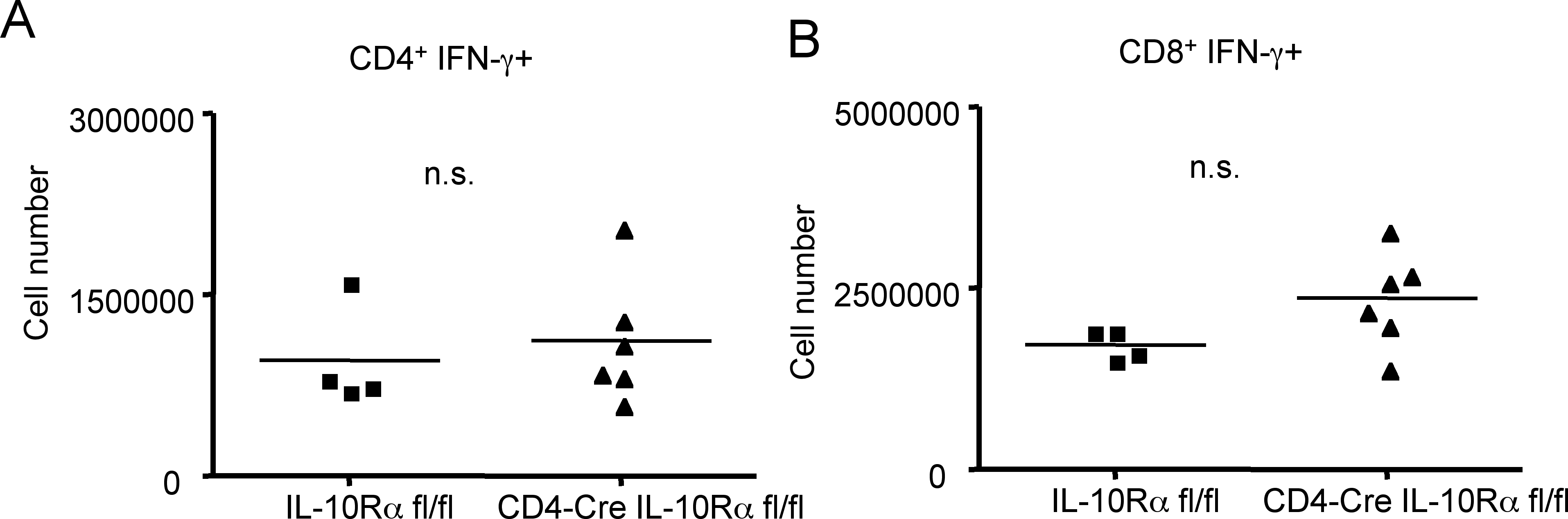

Supplement: Figure S4 — IL-10Rα deletion in T cells minimally affects effector T cell infiltration to the lung. (A, B) IL-10Rα fl/fl mice or CD4-Cre+ IL-10R fl/fl mice were infected with RSV. The numbers of total CD4+ T cells (A) or CD8+ (B) were determined by flow cytometry. P value was determined by unpaired two-tailed Student t test. n.s. non-significant. Data are representative of two independent experiments. (TIF) [file ppat.1002173.s004.tif]
